# New microsatellites for the Atacama Desert endemic *Balsamocarpon brevifolium* (Fabaceae)

**DOI:** 10.1002/aps3.11271

**Published:** 2019-06-11

**Authors:** Gonzalo Ravest, Pedro León‐Lobos, Carlos Aguirre, José Hernández, Gustavo Bolados, María Herminia Castro, Sergio Silva, Patricio Hinrichsen

**Affiliations:** ^1^ Instituto de Investigaciones Agropecuarias (INIA) La Platina Santa Rosa 11610 Santiago Chile; ^2^ INIA Intihuasi Camino a Peralillo s/n Vicuña Chile

**Keywords:** *Balsamocarpon brevifolium*, Caesalpinioideae, conservation genetics, Fabaceae, molecular markers, sustainable management

## Abstract

**Premise:**

Algarrobilla (*Balsamocarpon brevifolium*, Fabaceae) is an endemic xerophytic shrub restricted to the Atacama Desert in northern Chile. Extensive utilization of the region for coal production has endangered this species. Conservation efforts are underway, with genetic diversity analyses being key to the restoration of these populations.

**Methods and Results:**

Fifteen new microsatellite markers were developed for *B. brevifolium* and used to analyze three populations from the Atacama and Coquimbo regions in Chile. Microsatellites were highly polymorphic, with an average of 5.77 alleles per marker and an average level of expected heterozygosity of 0.72. These markers were evaluated and cross‐amplified on two related species (*Senna cumingii* and *Caesalpinia angulata*) with partial success.

**Conclusions:**

The development of this set of markers permits an extensive study of *B. brevifolium* populations for conservation purposes.

Algarrobilla (*Balsamocarpon brevifolium* Clos, Fabaceae) is a 1 to 2 m tall woody shrub endemic to Chile and with a restricted distribution between the south side of the Copiapó River (27°30′S) and north of the Elqui River (30°S), and from 300 to 2500 m elevation in the Andes Range. *Balsamocarpon brevifolium* is a conspicuous component of the arid region, particularly in the ecosystem of evergreen shrubs with microphyllous leaves that dominates the southern limits of the Atacama Desert of Chile (Luebert and Pliscoff, 2006). The arid climate is characterized by high temperatures and high solar radiation during the dry season in summer and scarce and scattered precipitation during winter (Aceituno et al., 1993). Every five to seven years, however, the El Niño Southern Oscillation carries intense precipitation to areas where *B. brevifolium* is present (Montecinos and Aceituno, 2003).

*Balsamocarpon brevifolium* frequently forms discontinuous populations, with small populations susceptible to alteration by human activities, and the species is under threat because of extensive charcoal production (Squeo et al., 2001, 2008; Estévez et al., 2010). In the past century, its yellow pods, which contain high tannin concentrations, were extensively harvested for use in the tanning industry (Wrann and Barros, 1987). Taking into account that ecological studies for this species are nonexistent, there is an urgent necessity to evaluate its genetic diversity and to understand the structure of different populations to design a conservation plan for this endangered species.

Molecular markers are optimal tools for identification of species and study of their population genetics, and may provide less laborious methods for a more accurate delimitation of species than phenotypic evaluations (Arif et al., 2010). Several types of molecular markers have been developed, including random‐amplified polymorphic DNA (RAPD), amplified fragment length polymorphism (AFLP), DNA barcoding, and single‐nucleotide polymorphism (SNP) markers. However, simple sequence repeats (SSRs or microsatellites) remain a powerful molecular marker because of their ubiquity in plant genomes, relative simplicity for analysis, high levels of intraspecific polymorphism, and the potential of amplifying in related species (Queller et al., 1993). Today, through massive sequencing of genomic DNA, it is possible to obtain a large number of these markers to be used in the characterization of species complexes. In this paper, we present the first SSR markers developed for *B. brevifolium* and demonstrate their cross‐amplification in related species, with the final aim of evaluating the genetic diversity of *B. brevifolium* through the study of the different populations of this endemic plant species of the Atacama Desert in Chile.

## METHODS AND RESULTS

Plant material was obtained from three different populations of *B. brevifolium* (Appendix 1; *n* = 21, *n* =18, and *n* =13 [only 13 plants were available for this population because of the poor preservation conditions of plant material]). The populations were selected based on the distinct geographic distribution of the species. Representative samples of each population were deposited at the Herbarium of La Serena University (ULS), La Serena, Chile (Appendix 1). Heavy deforestation and changing climatic conditions have decimated most populations of this species, making it difficult to obtain a larger number of samples per population. Plant material was also obtained from individuals of *Senna cumingii* (Hook. & Arn.) H. S. Irwin & Barneby (voucher ULS 4693) and *Caesalpinia angulata* (Hook. & Arn.) Baill. (ULS 4708) (Appendix 1), species that are related and cohabit with *B. brevifolium* (Luebert and Pliscoff, 2006), to test for the cross‐amplification of developed markers.

Total DNA was extracted from young leaves using the DNeasy Plant Mini Kit (QIAGEN, Hilden, Germany) following the manufacturer's instructions. DNA was quantified by using BioSpec‐nano (Shimadzu, Kyoto, Japan) and evaluated by electrophoresis on 0.8% agarose gels stained with ethidium bromide. DNA (50 μL, 450 ng/μL) from representative samples of *B. brevifolium* (vouchers ULS 14345, ULS 14346, ULS 14347; Appendix 1) was used for microsatellite identification at Ecogenics GmbH (Balgach, Switzerland). For this purpose, size‐selected fragments from genomic DNA were enriched for the formation of an SSR‐enriched library by using streptavidin‐coated magnetic beads and biotin‐labeled GATA and GTAT repeat oligonucleotides (Kijas et al., 1994; for a review, see Santana et al., 2009).

The SSR‐enriched library was analyzed on an Illumina MiSeq (Illumina, San Diego, California, USA) at Microsynth AG (Balgach, Switzerland) using the Nano 2 × 250 v2 format. The resulting 284,104 sequences were stitched and assembled using MIRA 4.0.1 software (Chevreux et al., 1999), and candidate microsatellites were searched in 22,361 paired‐end read assembled contigs using Finder version 4.09 (Benson, 1999). Of the candidate microsatellites, 5983 contigs contained a microsatellite insert with a tetra‐ or a trinucleotide of at least six repeat units or a dinucleotide of at least 10 repeat units. Finally, Primer3 (Rozen and Skaletsky, 1999) was used to design PCR primers for the selected microsatellites using standard default values. Suitable primer design was possible in 2564 microsatellite candidates. Of them, a total of 430 microsatellites were further analyzed with size ranges from 80 bp to 250 bp; di‐, tri‐, and tetranucleotide motifs were identified, with seven to 22 repetitions per motif. These 430 sequences were deposited in GenBank (accession numbers MH052690–MH053105 and MF136749–MF136763; see Table 1). From this set, we selected 40 microsatellite markers for evaluation on three populations of *B. brevifolium*.

**Table 1:** Microsatellites developed for *Balsamocarpon brevifolium*, an endemic xerophytic shrub from the Atacama Desert in Chile

Locus^a^	Primer sequences (5′–3′)	Repeat motif	Allele size range (bp)	GenBank accession no.
BBR‐005*	F: TGGCTCCTAAGTGTCCATGC	(GAA)_13_	190	MF136749
	R: CTCATTCTTTACGAAATTGCCCC			
BBR‐007*	F: TCGTACAGAATGAGGGACTAAAG	(AGA)_12_	135	MF136750
	R: CCTCCTTTTTACAAAATTGCCCC			
BBR‐008*	F: TGCAAGCATACCTCAGAAAGC	(TG)_12_	158	MF136751
	R: TCGATCATCACCTGCCACTC			
BBR‐009	F: CTGACGTCCATTTTCCCCTC	(TTC)_12_	150–190	MF136752
	R: GCAGCACCTCTGTTTTGGG			
BBR‐010	F: GTTCTACACACACTCACAGCG	(AGAA)_12_	175–220	MF136753
	R: TTTCCGACGTCCATTTTCCC			
BBR‐014	F: GCTTCCCGTCAATGCTCTTC	(CT)_11_	160–190	MF136754
	R: GCCACCCCAAGTGATTTTCC			
BBR‐017*	F: TGAAAGCTATGCTCTTTTCTTTACTG	(TCTT)_11_	170	MF136755
	R: GTCCCTTTCCATCGCAGTTG			
BBR‐018	F: GTCCCAAAAAGCCTAACTGTTTTC	(ATAC)_11_	170–215	MF136756
	R: GACTGTGGGTAGTTCATTATGGC			
BBR‐022	F: TCCGGATACTGTTCTGCGTC	(TCTT)_10_	120–130	MF136757
	R: CTCAACCGGCGATGAGAGG			
BBR‐025	F: GTGTTAGAGCTGATACATGAAATGC	(CATA)_10_	130–150	MF136758
	R: TGCCTTCTTTAATTCTGGTTTTTAG			
BBR‐026	F: AAAGTTGGGTCGCGAATGAC	(TTTC)_10_	170–178	MF136759
	R: CGCGATTTGATAACTATGCACC			
BBR‐039*	F: GCCCATTCATAAAAGAGTCACAAG	(AAG)_15_	237	MF136761
	R: TGCTTCTTCAATTCGCCGTC			
BBR‐041	F: ACGTACAGACATAGTGCTCAATC	(ACAT)_10_	190–200	MF136762
	R: AGCCGGGAAGATACTCATCG			
BBR‐043	F: TCCCTGAGTGTATCTGTCGC	(TATG)_10_	174–220	MF136763
	R: AGGCTGGTCAACTTTTGTGG			
BBR‐078	F: TGAGGGTTTTCATCATACCTGC	(AC)_17_	244–282	MH052718.1
	R: AACCTACCGATTGAAGGGGC			

a Annealing temperatures were the same for all loci (60°C).

*Monomorphic markers.

PCR amplifications contained, in a total volume of 12 μL, 30 ng of DNA, 2.4 μL of 5× colorless GoTaq Flexi buffer (Promega Corporation, Madison, Wisconsin, USA), MgCl_2_ 1 mM, 250 μM of dNTPs, 0.4 μM of primers, 0.5 units (0.1 μL) of GoTaq DNA Polymerase Flexi (Promega Corporation), and completed with distilled H_2_O. PCR cycling after an initial denaturation of 3 min at 94°C consisted of 35 cycles: 30 s at 95°C, annealing of 30 s at 60°C, and elongation of 60 s at 72°C. Finally, an incubation at 72°C for 10 min was included. PCR products were separated in 6% polyacrylamide gels and visualized by silver staining as described by Narváez et al. (2001).

Genetic diversity parameters including effective number of alleles (*A*_e_) and observed and expected levels of heterozygosity (*H*_o_ and *H*_e_, respectively) were estimated using GenAlEx 6.5 (Peakall and Smouse, 2012). For each SSR marker, *A*_e_ and *H*_e_ were estimated based on the frequency of each allele per population, and *H*_o_ corresponded to the frequency of heterozygous individuals calculated for each population.

Of the 40 primer sets analyzed, 15 markers (10 polymorphic and five monomorphic) that showed clear amplification were analyzed in more detail (Table 1). Twenty‐five primers showed no amplification, or a complex pattern, and were not further analyzed. Sequences of the 15 markers that showed clear amplification have been deposited in GenBank (Table 1); sequences of the remaining 25 primers are available upon request. For the 10 polymorphic primers, allele number ranged from three to nine (average of 5.77 alleles per marker in the populations), *A*_e_ ranged from 2.27 to 6.00, and the expected and observed levels of heterozygosity varied from 0.56 to 0.83 and from 0.53 to 1.00, respectively (Table 2). These are the first polymorphic microsatellite markers developed for *B. brevifolium*, although not the first genetic markers from Leguminosae applied to the characterization of this species (Gagnone et al., 2016).

**Table 2:** Genetic properties of 10 polymorphic microsatellites developed for *Balsamocarpon brevifolium* characterized in three populations from the Atacama and Coquimbo regions, Chile.^a^

Locus*	Population 1 (*n* = 21)				Population 2 (*n* = 18)				Population 3 (*n* = 13)			
	*A*	*A*_*e*_	*H*_*e*_	*H*_*o*_	*A*	*A*_*e*_	*H*_*e*_	*H*_*o*_	*A*	*A*_*e*_	*H*_*e*_	*H*_*o*_
BBR‐009	6	3.76	0.73	0.95	5	3.45	0.71	1	3	2.27	0.56	0.53
BBR‐010	7	3.76	0.73	0.92	6	4.55	0.78	0.94	6	4.44	0.77	0.77
BBR‐014	6	4.55	0.78	0.67	7	4.59	0.78	0.89	7	6	0.83	0.85
BBR‐018	9	3.82	0.74	0.9	8	4.03	0.75	0.78	4	2.71	0.63	0.77
BBR‐022	3	2.37	0.58	0.9	3	2.32	0.57	0.94	3	2.98	0.66	0.85
BBR‐025	5	3.97	0.75	1	5	3.43	0.71	1	7	5.23	0.81	1
BBR‐026	7	3.72	0.73	0.71	5	2.84	0.65	0.83	4	2.72	0.63	0.92
BBR‐027	7	5.1	0.8	0.67	8	4.27	0.77	0.72	6	4.57	0.78	1
BBR‐041	6	3.93	0.75	1	6	3.34	0.7	0.89	4	3.12	0.68	1
BBR‐043	8	5.08	0.8	0.95	6	3.3	0.69	1	8	5.54	0.82	1

Note

*A* = number of alleles; *A*_e_ = number of effective alleles; *H*_e_ = expected heterozygosity; *H*_o_ = observed heterozygosity; *n* = number of individuals.

a Locality and voucher information are provided in Appendix 1.

*Information is provided only for the 10 markers identified as polymorphic.

The new markers also amplified in *C. angulata*, a species belonging to the same tribe (Caesalpinieae), and in *S. cumingii* (caper shrub), a species within a different tribe (Cassieae; Ulibarri, 2008) of the Caesalpinioideae subfamily (LPWG, 2017). However, results showed only partial amplification of the new markers in *S. cumingii* and less amplification in *C. angulata* (Table 3).

**Table 3:** Transferability of 10 polymorphic SSR markers developed for *Balsamocarpon brevifolium* in two Atacama Desert shrubs, *Caesalpinia angulata* and *Senna cumingii* (Fabaceae).^a^

Locus	*Caesalpinia angulata*					*Senna cumingii*					*Balsamocarpon brevifolium*
	1	2	3	4	5	1	2	3	4	5	
BBR‐009	+/−	+/−	+/−	+/−	+/−	+/−	+/−	+/−	+/−	+/−	++
BBR‐010	—	—	—	—	—	—	—	—	—	—	++
BBR‐014	115–170	+	+	+	+	+	+	+	+	+	+
BBR‐018	—	—	+/−	–	+/−	210	++	++	++	++	++
BBR‐022	+/−	+/−	+/−	+/−	+/−	190	+	+	+	+	+
BBR‐025	—	—	—	—	—	—	—	—	—	—	++
BBR‐026	+/−	+/−	—	+/−	—	+/−	+/−	+/−	+/−	+/−	+
BBR‐041	—	—	—	—	—	174	++	++	++	++	++
BBR‐043	+/−	+/−	+/−	+/−	+/−	+/−	+/−	+/−	+/−	+/−	++
BBR‐078	+/−	+/−	+/−	+/−	+/−	+/−	+/−	+/−	+/−	+/−	++

Note

++ = clear and strong PCR signal; + = good signal; +/− = weak signal; — = no amplification.

a Locality and voucher information are provided in Appendix 1.

The estimated allele sizes for each primer/species combination are indicated for sample 1 of each species.

## CONCLUSIONS

We have identified 15 new microsatellite markers for algarrobilla, 10 of which had high levels of polymorphism, representing the first markers developed in *B. brevifolium*. Some of these markers were also useful in the related species *C. angulata* and *S. cumingii*, which share the same habitat as *B. brevifolium*. Analyses of *B. brevifolium* individuals from different locations in the Atacama and Coquimbo regions in Chile would help characterize algarrobilla populations for conservation purposes and be the basis for future genetic studies for this species and possibly other endangered and related legumes endemic to northern Chile.

## Data Availability

Sequence information for the developed primers has been deposited to the National Center for Biotechnology Information (NCBI); GenBank accession numbers are provided in Table 1.

## APPENDIX 1 Herbarium vouchers and geographical coordinates for representative specimens of *Balsamocarpon brevifolium*,* Caesalpinia angulata*, and *Senna cumingii* from Chile used for the development of microsatellite markers.^a^

**Table:**

Species	Population code	Voucher no.^b^	Geographical coordinates	Elevation (m)	*N*
*Balsamocarpon brevifolium* Clos	Population 1	ULS 14346	28°13′S, 70°42′W	426	21
	Population 2	ULS 14345	28°53′S, 70°46′W	1043	18
	Population 3	ULS 14347	28°08′S, 70°51′W	1096	13
*Caesalpinia angulata* (Hook. & Arn.) Baill.	ND	ULS 4693	29°7′S, 70°53′W	750	>10
*Senna cumingii* (Hook. & Arn.) H. S. Irwin & Barneby	ND	ULS 4708	28°38′S, 70°49′W	435	>10

Note

*N* = number of individuals; ND = population not defined.

a GPS and elevation ranges for all individuals within each population: Population 1 = 28°5′S, 70°36′W to 28°13′S, 70°43′W, 399–540 m; Population 2 = 28°53′S, 70°32′W to 28°55′S, 70°51′W, 863–2130 m; Population 3 = 29°8′S, 70°36′W to 29°11′S, 70°52′W, 1091–1999 m.

b Voucher specimens were deposited at the herbarium of Universidad de La Serena (ULS), La Serena, Chile.
